# A Pessary Removed from the Vagina after Having Been Worn Eleven Years

**Published:** 1884-04

**Authors:** Thomas L. Raines

**Affiliations:** Atlanta, Ga.


### A PESSARY REMOVED FROM THE VAGINA AFTER HAVING BEEN WORN ELEVEN YEARS.

BY THOMAS L. RAINES, M. D., ATLANTA, GA.

March 9th I was called to see Mrs. B., widow, who gave the following history: I was treated in 1873 by a physician in New Orleans for womb disease. He said the womb was displaced, and that I would have to wear an instrument to keep it in place. He applied the instrument, and for a time it gave me very great relief. After a few months, however, I began to suffer again, and have suffered ever since. In November last my suffering became so great I called in a physician, who gave me a wash and instructed me to use it for a few days, saying that he would call again. This he did not do. I have had no other treatment since I left New Orleans, and, so far as I know, the instrument has never been removed.

Assisted by Dr. C. A. White, the patient was etherized and a digital examination made. Just within the vaginal orifice I found a hard substance which was completely imbedded in the mucous tissues. Remembering the history of the patient, I at once thought of the pessary that had been introduced eleven years before, and determined to cut through this hardened mucous membrane. I had very little difficulty in exposing and removing a hard rubber pessary. Some slight hemorrhage followed its removal. The patient was instructed to use the hot vaginal douche, which she did, and is now about well; all her suffering disappearing very soon after the pessary was removed.

